# Isolated traumatic aniridia with full and partial iris expulsion in pseudophakic eyes

**DOI:** 10.1186/s12886-022-02615-4

**Published:** 2022-10-01

**Authors:** Sho Ichioka, Akiko Ishida, Kazunobu Sugihara, Ichiya Sano, Masaki Tanito

**Affiliations:** Department of Ophthalmology, Shimane University Faculty of Medicine, 89-1 Enya, Izumo, Shimane, 693-8501 Japan

**Keywords:** Blunt trauma, Expulsive injury, Fall, Iris dialysis, Small incisional cataract surgery, Intraocular lens

## Abstract

**Background:**

Total aniridia after ocular trauma without disruption of the intraocular lens (IOL) has been reported in patients with a history of small-incisional cataract surgery. We report one case each of total and partial aniridia after accidental falls experienced by two elderly Japanese women.

**Case presentations:**

Case 1. A 76-year-old woman with a history of small-incisional cataract surgery more than 10 years previously fell onto concrete and had a contusion that affected the left side of her face. At the initial visit, the best-corrected visual acuity (BCVA) was hand motions and the intraocular pressure (IOP) was 38 mmHg in her left eye (OS). A blood clot was present in the well-formed anterior chamber and expulsed iris tissue was seen beneath the conjunctiva. Exploratory surgery showed no scleral laceration other than the previous sclerocorneal tunnel. After hyphema removal, total aniridia and an intact in-the-bag fixed IOL were seen. By 4 months, the BCVA was 1.2 and the IOP was 13 mmHg OS. Case 2. An 88-year-old woman with a history of small-incisional cataract surgery more than 10 years previously had a fall that resulted in right-sided zygomatic and maxillary bone fractures. The BCVA was light perception and the IOP was 29 mmHg in her right eye (OD). Exploratory surgery showed no scleral laceration and the previous sclerocorneal tunnel was found; iris strand prolapsing from the sclerocorneal tunnel was seen. After hyphema removal, partial iris loss and an intact lens position were seen. By 1 week postoperatively, the BCVA was 0.05 OD and the IOP was 12 mmHg OD.

**Conclusions:**

It has been postulated that previously created small-incision tunnels can function as release valves during blunt trauma by preventing further global rupture and limiting IOL prolapse or retinal injury. Our cases suggested this can happen even long periods after cataract surgery. The case with partial aniridia demonstrated the process of the expulsive aniridia, and its findings do not contradict the postulated mechanisms.

**Supplementary Information:**

The online version contains supplementary material available at 10.1186/s12886-022-02615-4.

## Background

Many cases of isolated traumatic aniridia, i.e., aniridia leaving an intact in-the-bag fixed intraocular lens (IOL) have been reported in patients with a history of small-incisional cataract surgery [1–19]; all of these eyes were reported to have complete aniridia. We report one case each of total and partial aniridia after accidental falls by two elderly Japanese women. Our cases suggested traumatic aniridia can happen even long periods after cataract surgery. In addition to this, based on the observed findings, we discussed the underlying mechanisms of this ocular trauma.

## Case presentations

**Fig. 1:**
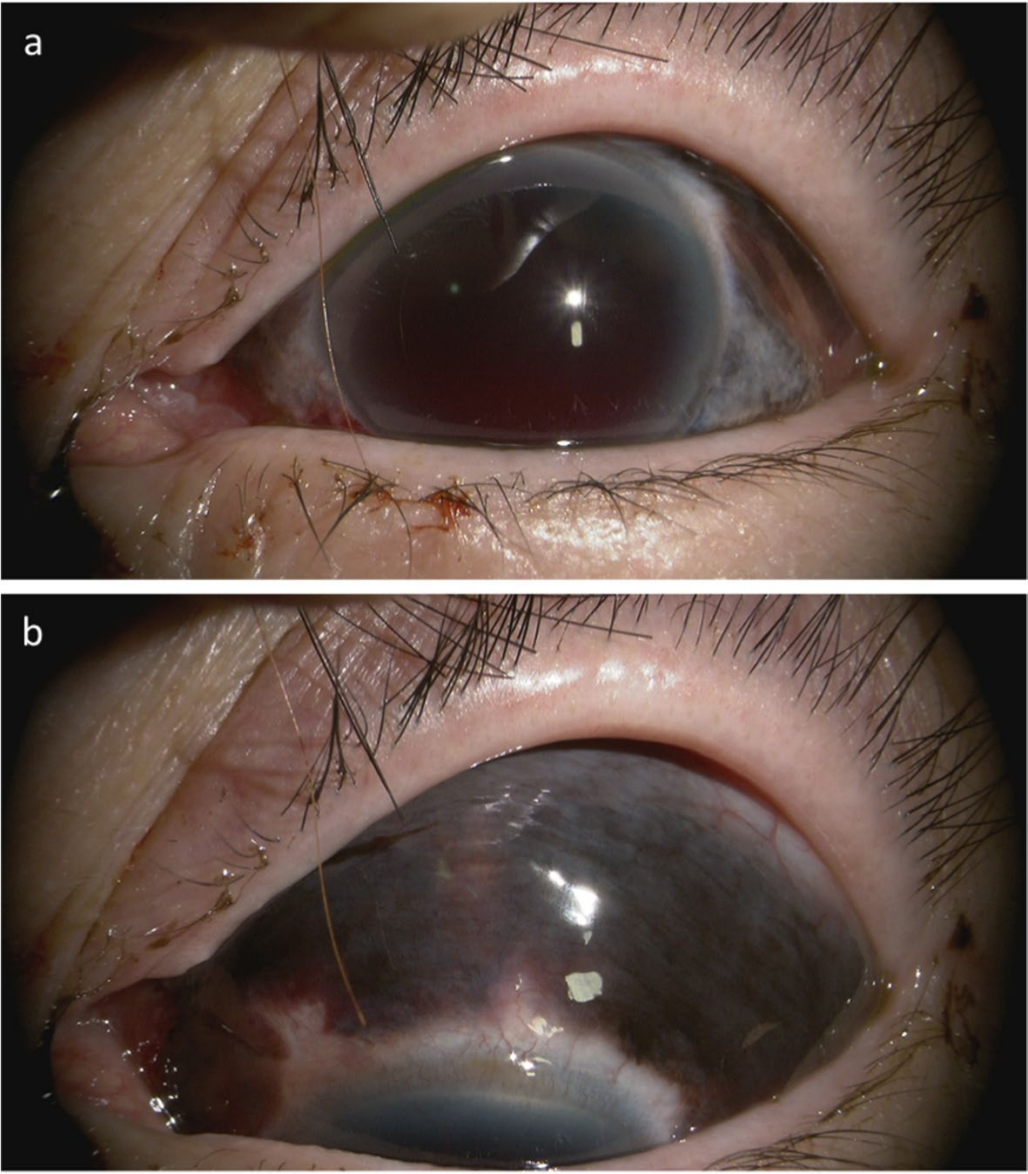
Case 1, OS. Anterior-segment findings at the initial visit on the day of injury. **a** Due to hyphema, the iris and IOL are not visible. **b** In the superior hemisphere, brown tissue is seen beneath the conjunctiva

**Fig. 2:**
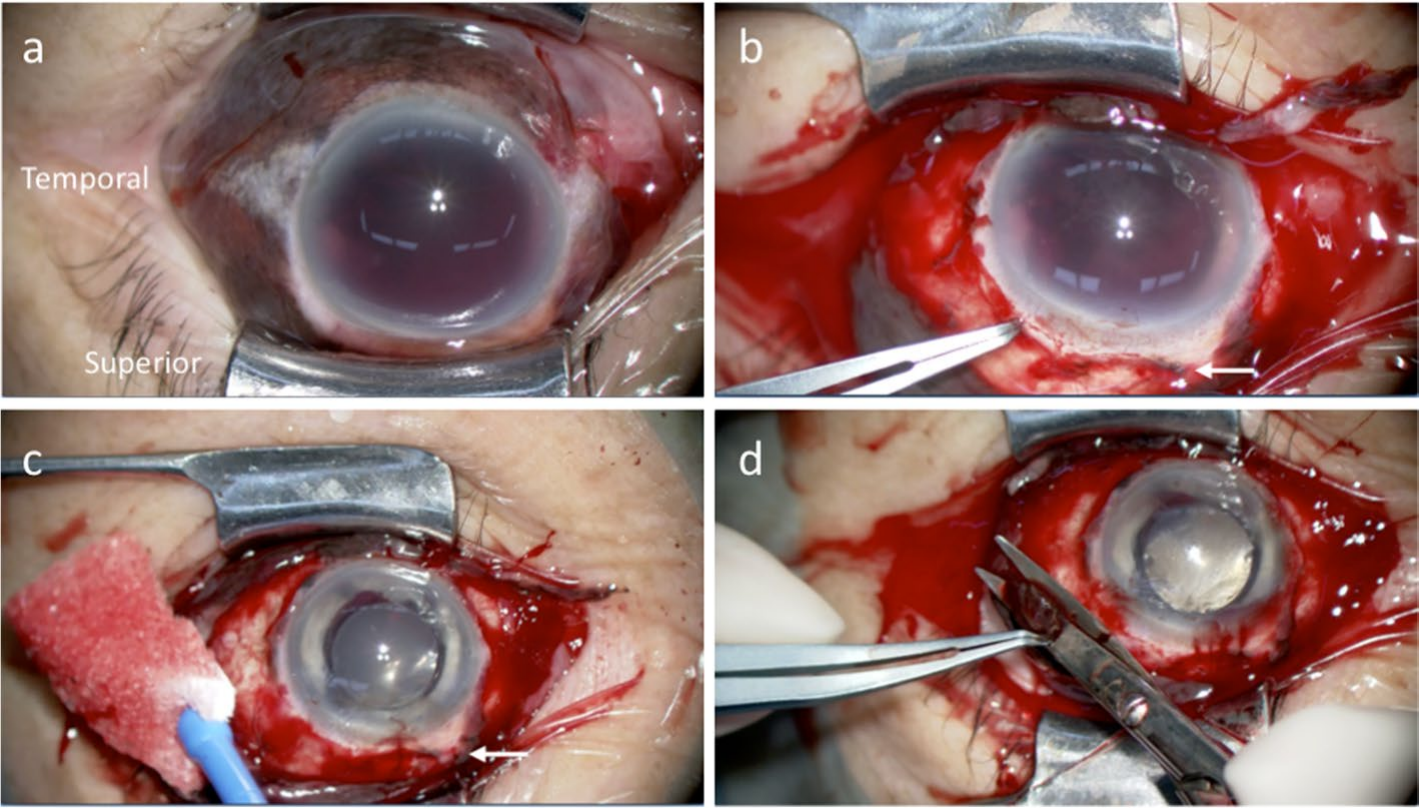
Case 1, OS. Intraoperative findings of surgery performed on the same day of injury. **a** Subconjunctival brown tissue is seen all around the eye. **b** A circumferential peritomy reveals no evidence of scleral laceration other than previous sclerocorneal tunnel (arrow). **c** Total aniridia is revealed after hyphema removal by bimanual irrigation and aspiration. Interrupted sutures are placed at the sclerocorneal tunnel (arrow). **d** Iris tissue beneath the conjunctiva is removed as much as possible by excising with Tenon’s capsule

**Fig. 3:**
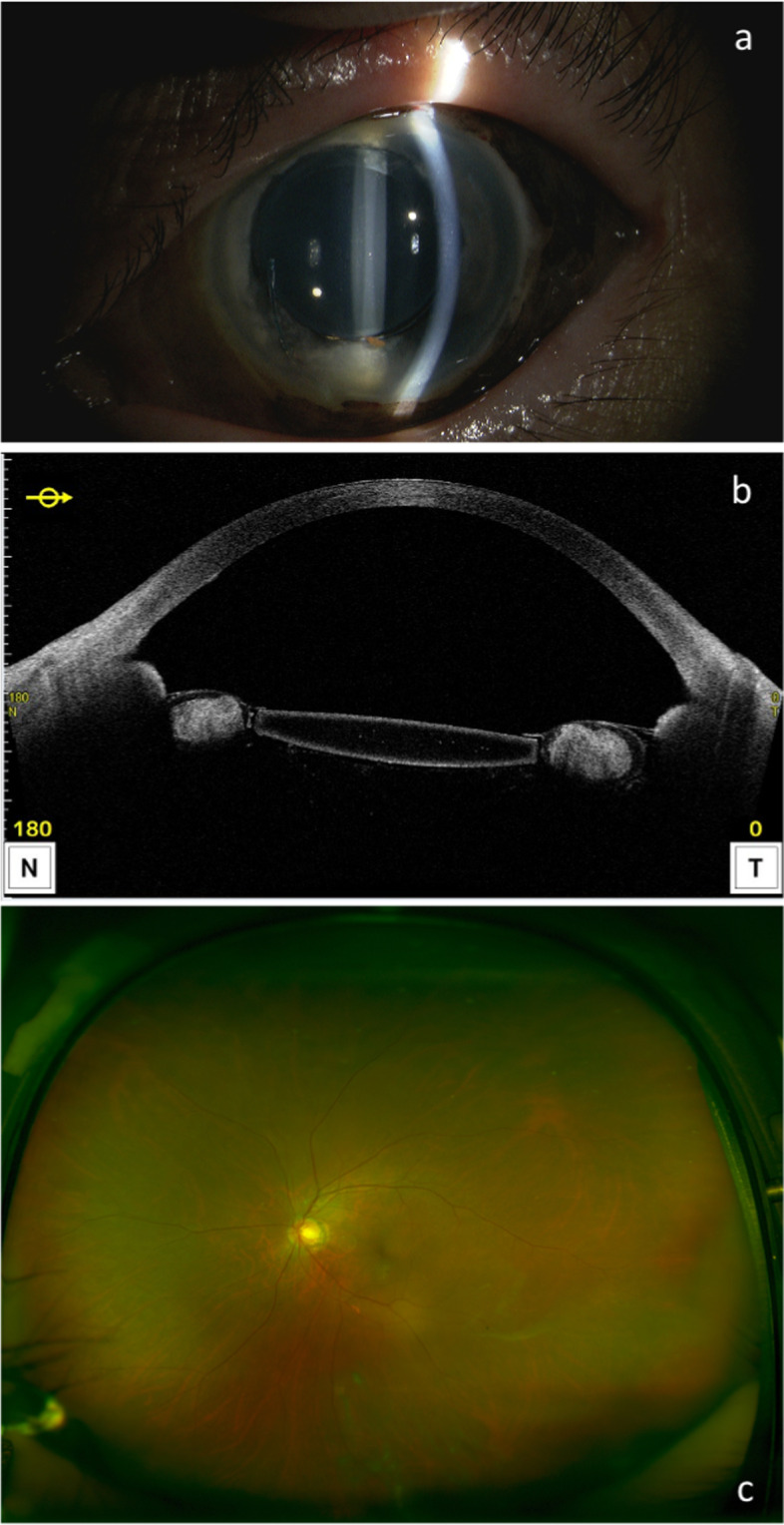
Case 1, OS. One-month postoperative findings. **a**, **b** Slit-lamp examination and anterior-segment OCT reveal complete iris loss and intact lens position. **c** Wide-field fundus photograph reveals no vitreoretinal injury

Case 1 A 76-year-old woman fell onto concrete and suffered a stroke that affected the left side of her face. On the same day, she was referred to her local ophthalmologist and presented to ophthalmology department of Shimane University Hospital immediately due to a suspected left global rupture. Based on the information provided by the patient, she had a history of uncomplicated bilateral phacoemulsification cataract surgeries and intraocular lens (IOL) implantations more than 10 years previously, but the surgical details were unavailable. At the initial visit to our department, the best-corrected visual acuity (BCVA) measured by decimal visual acuity chart was 1.2 in her right eye (OD) and hand motions in her left eye (OS); the intraocular pressures (IOPs) were 13 mmHg OD and 38 mmHg OS. Slit-lamp examination showed a blood clot in the well-formed anterior chamber; the iris and IOL were not observable (Fig. 1a). Brown-colored tissue was seen beneath the conjunctiva (Fig. 1b). The presence of a relative afferent pupillary reflex was unknown due to dense hyphema OS. B-mode echography (UD-800, Tomey Corporation, Nagoya, Japan) showed neither dense vitreous hemorrhage nor retinal detachment. The patient underwent an exploratory surgery on the same day (Fig. 2a-d, Video 1). Intraoperatively, a circumferential peritomy showed no evidence of scleral laceration other than the previous sclerocorneal tunnel (Fig. 2b, arrow). After hyphema removal by bimanual irrigation and aspiration, total aniridia was seen (Fig. 2c). Iris tissue beneath the conjunctiva was removed by excising Tenon’s capsule (Fig. 2d); the conjunctiva then was secured and the surgery was finished. Postoperatively, topical antibiotics (1.5% levofloxacin, Pfizer Japan Inc., Tokyo, Japan) 4 times daily were prescribed for 1 month and stopped; topical steroids (0.1% betamethasone, 0.1% betamethasone, Santen Pharmaceutical, Osaka, Japan) 4 times daily were prescribed for 2 months, followed by twice daily for another 2 months, and then stopped. A postoperative slit-lamp examination and anterior-segment optical coherence tomography (OCT, Casia 2, Tomey Corporation) showed complete iris loss and an intact intracapsular-fixed lens position (Fig. 3a, b). Wide-field fundus photography (Optos 200Tx, Nikon, Japan) showed no vitreoretinal injury (Fig. 3c). At the final visit 4 months postoperatively, the BCVA was 1.2 bilaterally and the right and left IOPs were 16 and 13 mmHg, respectively; no evidence of sympathetic ophthalmia was observed during the follow-up period.

**Fig. 4:**
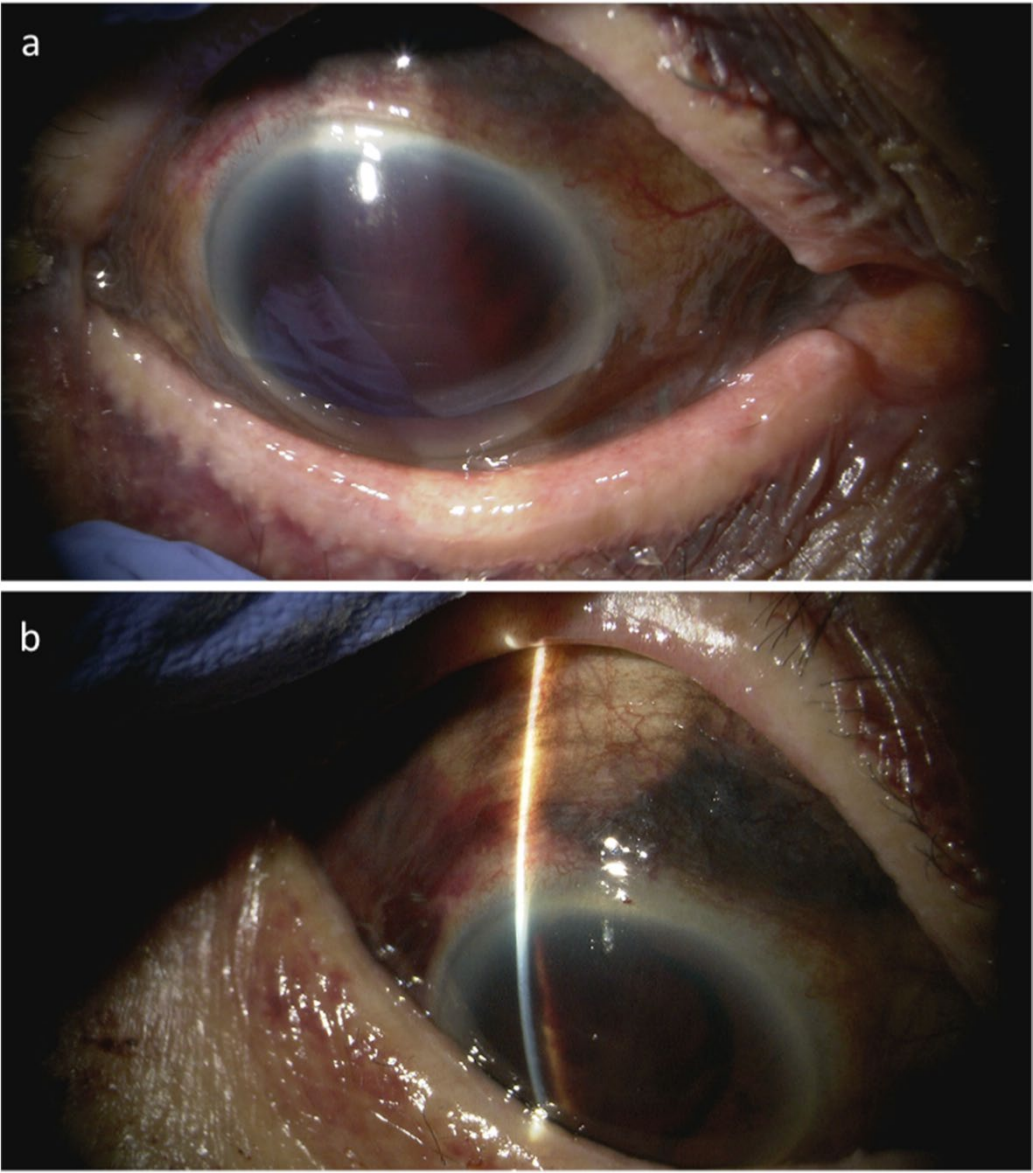
Case 2, OD. Anterior segment findings after the injury. **a** Four days after the injury, the iris and IOL are not seen due to dense hyphema. **b** Ten days after the injury, brown tissue is seen beneath the conjunctiva in the superior hemisphere

**Fig. 5:**
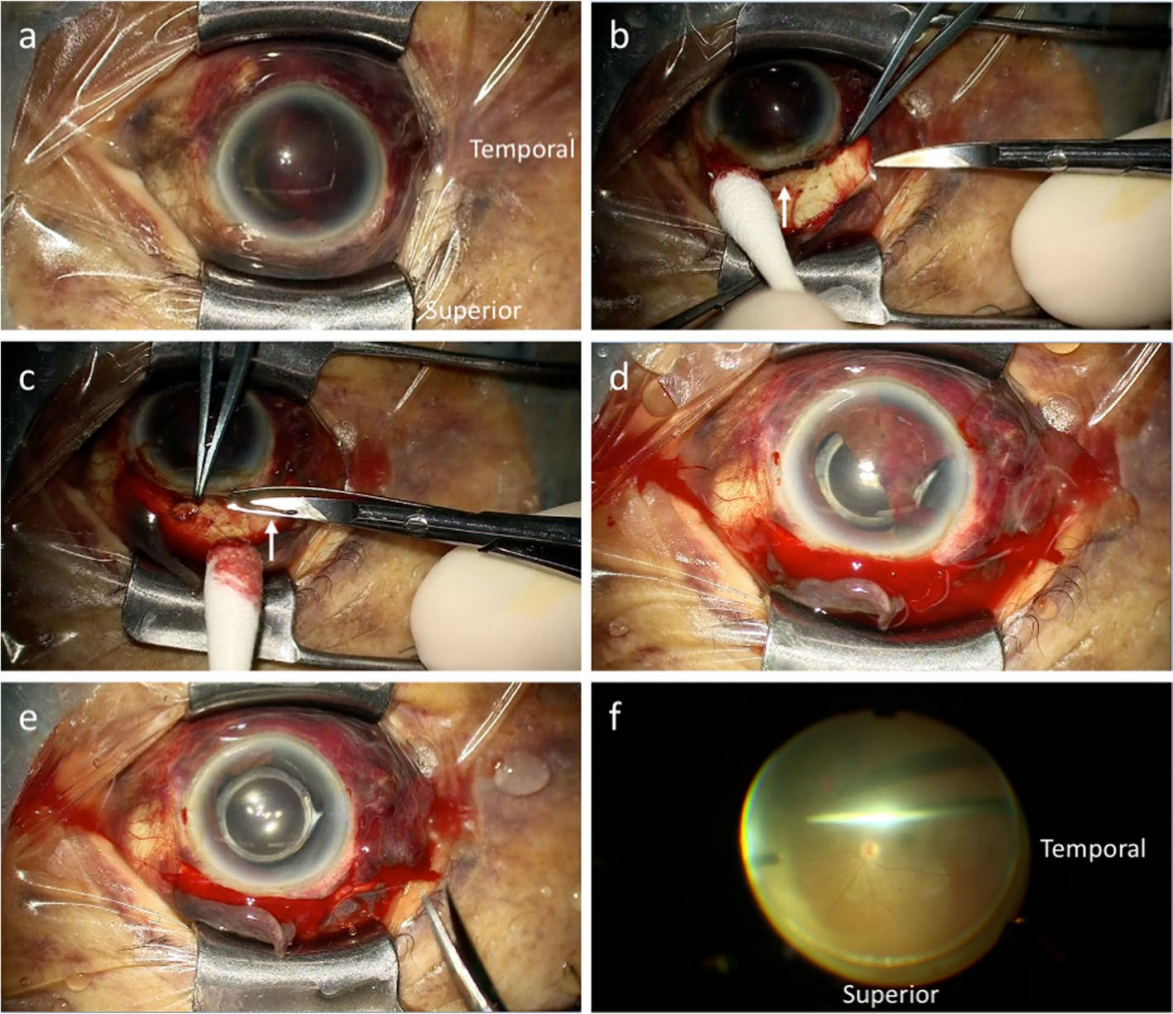
Case 2, OD. Intraoperative findings performed 11 days after the injury. **a** Subconjunctival brown tissue is seen in the superior and nasal quadrants of the eye. **b** A superior limbal peritomy shows no evidence of scleral laceration other than the previous sclerocorneal tunnel (arrow). **c** Iris strand prolapsing from the sclerocorneal tunnel is seen (arrow). **d**, **e** Partial iridodialysis and iris defect temporally are seen after hyphema removal using a vitreous cutter. **f** No fundus injury is seen after the removal of the vitreous hemorrhage during pars plana vitrectomy

**Fig. 6:**
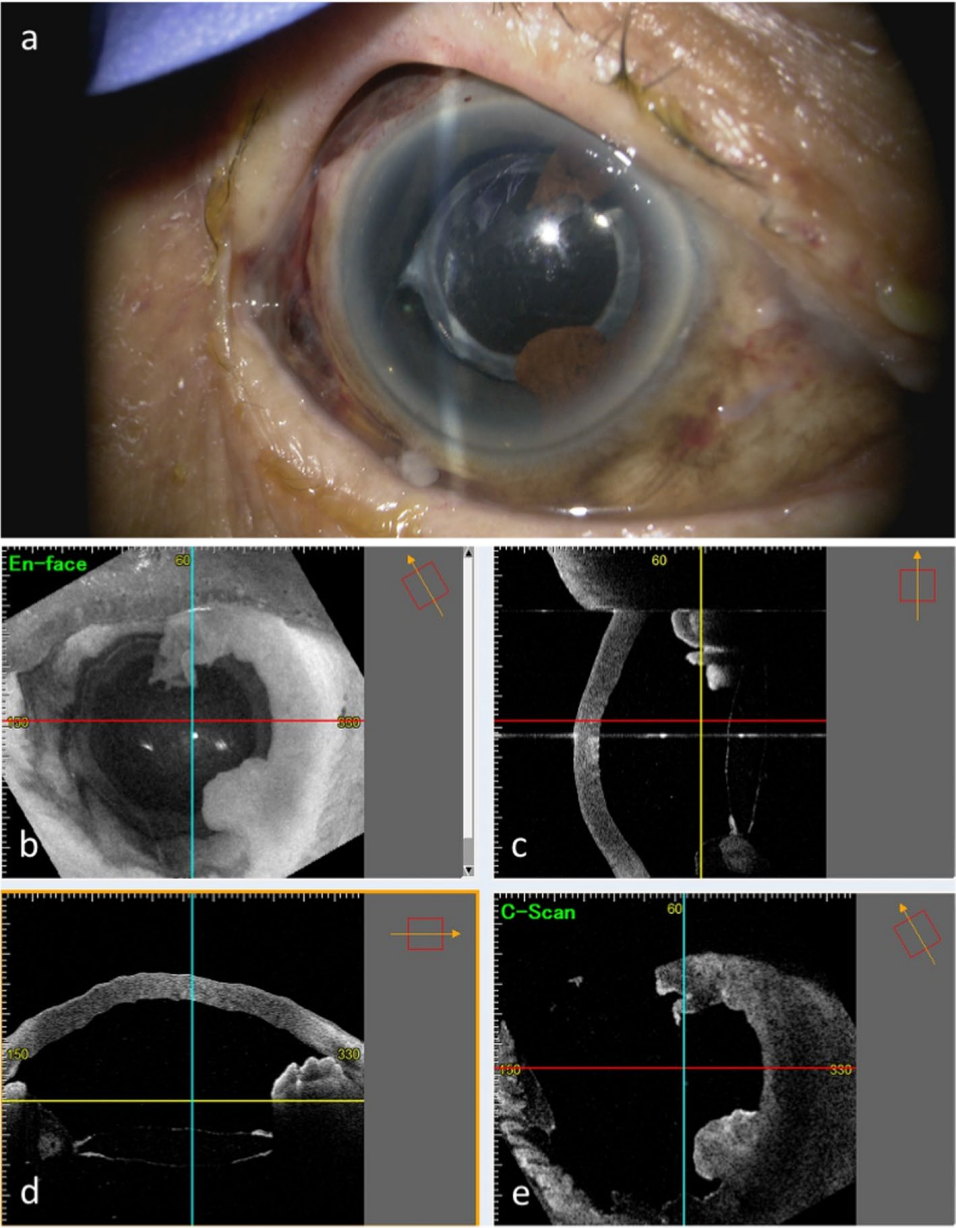
Case 2, OD. One-week postoperative findings. Slit-lamp examination (**a**) and anterior-segment OCT (**b-e**) show partial iris loss temporally and an intact lens position. **b** En-face, (**c**) vertical scan, (**d**) horizontal scan, and (e) C-scan images of anterior-segment OCT are shown

Case 2 was that of an 88-year-old woman who fell in her room and injured the right side of her face, although she could not recall the details. On the same day, she was referred to her local emergency room and then to emergency room of Shimane University Hospital immediately due to the right-sided zygomatic and maxillary bone fractures. Four days later, she presented to ophthalmology department of Shimane University Hospital because of decreased vision OD. Based on the information provided by the patient, she had a history of bilateral uncomplicated phacoemulsification cataract surgery and IOL implantation more than 10 years previously, but the surgical details were unavailable. At the initial visit to our department, the right and left BCVAs were light perception and 0.8 by decimal visual acuity chart, respectively; the respective IOPs were 29 and 16 mmHg. Slit-lamp examination showed a blood clot in the well-formed anterior chamber; the iris and IOL were not observable (Fig. 4a). Brown-colored tissue was seen beneath the conjunctiva (Fig. 4b). The presence of a relative afferent pupillary reflex was unknown due to dense hyphema OD. B-mode echography showed a vitreous hemorrhage but no retinal detachment. Helical computed tomography (Aquilion ONE, Toshiba Medical Systems, Tochigi, Japan) showed no evidence of an optic canal fracture. Topical dorzolamide and timolol were prescribed twice daily. Eight days after the injury, she underwent surgery for a blowout fracture. Since the vision did not recover, an exploratory surgery was performed 11 days after the injury (Fig. 5a-f, Video 2). Intraoperatively, a superior limbal peritomy showed no evidence of scleral laceration other than the previous sclerocorneal tunnel (Fig. 5b, arrow); iris strand prolapsing from the sclerocorneal tunnel was seen (Fig. 5c, arrow). After hyphema removal using a vitreous cutter, a partial iridodialysis and iris defect in the temporal side were seen (Fig. 5d, e). After the vitreous hemorrhage was removed during pars plana vitrectomy, no fundus injury was seen (Fig. 5f). Iris tissue beneath the conjunctiva was removed, the conjunctiva was secured, and surgery was finished. Postoperatively, topical antibiotics (1.5% levofloxacin, Pfizer Japan Inc.,) and steroid (Sanbetason, Santen Pharmaceutical) 4 times daily were prescribed. At the final visit 1 week postoperatively, the right and left BCVAs were 0.05 and 0.5, respectively; the respective IOPs were 12 and 7 mmHg; and slit-lamp examination and anterior-segment OCT (Casia 2) (Fig. 6a-e) showed partial iris loss in the temporal side and an intact lens position. She was referred to her local hospital for follow-up observation.

## Discussion and conclusions

We conducted a literature search and, including the subjects in this report, found 22 eyes of 21 subjects (6 men, 15 women) with isolated traumatic aniridia after small-incisional cataract surgery (Table 1) [1–19]. Considering that most of the population with a history of cataract surgery are elderly, with some exceptions, the reported patients were old and accidental falls were the most frequent cause of this rare ocular trauma. Although one case of non-expulsive aniridia was reported [6], traumatic total aniridia usually accompanies expulsion of dialyzed iris. Based on the type of incision created in the previous surgery, expulsed iris tissue can be found beneath the conjunctiva if a previous surgery was performed via a sclerocorneal tunnel as in the current cases, and the iris tissue can be lost (i.e., vanishing iris [3]) if the previous surgery was performed via clear corneal incision. Fortunately, including our cases, we could not find any cases in the literature of sympathetic ophthalmia after this type of trauma. While age-related decline in immune-reaction and/or use of topical steroid can explain this, although this speculation is inconclusive.

**Table 1:** Reported cases of isolated traumatic aniridia after small incisional cataract surgery

Age (years)	Sex	Eye	Incision	Position	Width (mm)	Trauma	Iris expulsion	Year	First author
68	F	NA	SCI	Superior	5.0	Fell and hit dresser	Total	1997	Navon [1]
82	F	OD	SCI	Superior	5.25	Fell and hit cabinet edge	Total	1999	Lim [2]
91	F	OD	CCI	Temporal	4.0	Fell and hit table edge	Total	2002	Ball [3]
91	M	OD	CCI	Superior	4.1	Fell and hit table edge	Total	2003	Blomquist [4]
84	F	OD	CCI	Temporal	3.2	Fell onto concrete	Total	2004	Walker [5]
79	F	OD	NA	Superior	3.5–4.0	Fell and hit toilet seat	Non-expulsive total aniridia	2004	Sullivan [6]
53	M	OD	CCI	Temporal	3.0	Motor vehicle accident	Total	2005	Kahook [7]
85	F	OS	CCI	Superior	NA	Fell onto pavement	Total	2006	Muzaffar [8]
74	F	OS	CCI	Temporal	4.0	Fell	Total	2006	Sheth [9]
45	M	OS	CCI	Superotemporal	3.5	Hit car door	Total	2006	Doro [10]
45	M	OS	CCI	Temporal	NA	Fell	Total	2007	Parmeggiani [11]
79	F	OS	CCI	Superotemporal	3.2	Fell and hit curb	Total	2007	Prabhu [12]
76	F	OS	CCI	Temporal	2.75	Fell	Total	2009	Georgalas [13]
56	F	OS	CCI	Superotemporal	NA	Fell	Total	2011	Zurutuza [14]
66	F	OS	CCI	Temporal	3.0	Hit shelf	Total	2012	Mikhail [15]
87	F	OD	CCI	NA	2.85	Fell onto concrete	Total	2012	Oltra [16]
		OS	CCI	NA	3.2	Fell onto concrete	Total		
56	M	OS	OCCI	Inferotemporal	3.0	Hit desk edge	Total	2013	Eom [17]
64	F	OS	CCI	NA	3.0	Fell and hit step edge	Total	2016	Sophocleus [18]
74	M	NA	CCI	NA	NA	Blunt trauma	Total	2017	Ruiz-Mederano [19]
76	F	OS	SCI	Superior	NA	Fell onto concrete	Total	2022	Current report
88	F	OD	SCI	Superior	NA	Fell	Partial		

*F* Female, *M* Male, *SCI* Sclerocorneal incision, *CCI* Clear corneal incision, *OCCI* Opposite CCI, *NA* Not available

All reported cases of traumatic aniridia were complete (Table 1); thus, the partial expulsion in this report is unique in the literature. The following scenarios were postulated as mechanisms of traumatic aniridia [1–3]: 1) force exerted on the globe elevates IOP, 2) a previously created self-sealing cataract incision opens and iris extrudes through the incision, 3) the iris stuck in the incision is avulsed from the iris root in turn from proximal to the incision by the elevated IOP, 4) depressurization prevented extension of the wound and the posterior structures were held back by the in-the-bag fixed IOL, 5) the impact ended and the anterior chamber reformed by the self-sealing incision. Thus, a small-incision sclerocorneal or clear corneal tunnel can function as a release valve during blunt trauma and prevent limbal or extraocular muscle insertion rupture, limiting IOL prolapse or retinal injury. In case 2, the iris strand prolapsing from the sclerocorneal tunnel observed intraoperatively can be a snapshot of step 3.

Both of the current cases suggested that expulsion of iris tissue through a previously created small incision can happen even long periods after cataract surgery. The case with partial aniridia demonstrates the process of the expulsive aniridia, and its findings do not contradict the previously postulated mechanisms.

## Supplementary information

**Additional file 1. **Case 1. Surgical video.

**Additional file 2. **Case 2. Surgical video.

## Data Availability

All data generated or analyzed during this study are presented in this article. Further enquiries can be directed to the corresponding author.

## Abbreviations

IOL: Intraocular lens
BCVA: Best-corrected visual acuity
IOP: Intraocular pressure
OS: Left eye
OD: Right eye

## References

[1] Navon SE (1997). Expulsive iridodialysis: An isolated injury after phacoemulsification. J Cataract Refract Surg.

[2] Lim JI, Nahl A, Johnston R, Jarus G (1999). Traumatic total iridectomy due to iris extrusion through a self-sealing cataract incision. Arch Ophthalmol.

[3] Ball J, Caesar R, Choudhuri D (2002). Mystery of the vanishing iris. J Cataract Refract Surg.

[4] Blomquist PH (2003). Expulsion of an intraocular lens through a clear corneal wound. J Cataract Refract Surg.

[5] Walker NJ, Foster A, Apel AJ (2004). Traumatic expulsive iridodialysis after small-incision sutureless cataract surgery. J Cataract Refract Surg.

[6] Sullivan CA, Murray A, McDonnel P (2004). The long-term results of nonexpulsive total iridodialysis: an isolated injury after phacoemulsification. Eye (Lond).

[7] Kahook MY, May MJ (2005). Traumatic total iridectomy after clear corneal cataract extraction. J Cataract Refract Surg.

[8] Muzaffar W, O'Duffy D (2006). Traumatic aniridia in a pseudophakic eye. J Cataract Refract Surg.

[9] Sheth HG, Laidlaw AH (2006). Traumatic aniridia after small incision cataract extraction. Cont Lens Anterior Eye.

[10] Doro D, Deligianni V (2006). Ultrasound biomicroscopy in traumatic aniridia 2 years after phacoemulsification. J Cataract Refract Surg.

[11] Parmeggiani F, Mantovani E, Costagliola C, Campa C, Steindler P (2007). Total aniridia after nonperforating trauma of a pseudophakic eye: ultrasound biomicroscopic findings. J Ultrasound Med.

[12] Prabhu A, Nayak H, Palimar P (2007). Traumatic expulsive aniridia after phacoemulsification. Indian J Ophthalmol.

[13] Georgalas I, Petrou P, Chauhan D, Papaconstantinou D, Ladas I (2009). Posttraumatic aniridia following phacoemulsification. Can J Ophthalmol.

[14] Zurutuza A, Andonegui J, Berástegui L (2011). Traumatic expulsive iridodialysis with vitreous prolapse. Int Ophthalmol.

[15] Mikhail M, Koushan K, Sharda RK, Isaza G, Mann KD (2012). Traumatic aniridia in a pseudophakic patient 6 years following surgery. Clin Ophthalmol.

[16] Oltra EZ, Chow CC, Lunde MW (2012). Bilateral traumatic expulsive aniridia after phacoemulsification. Middle East Afr J Ophthalmol.

[17] Eom Y, Kang SY, Song JS, Kim HM (2013). Traumatic aniridia through opposite clear corneal incision in a pseudophakic eye. J Cataract Refract Surg.

[18] Sophocleous S. Doctor, where is my iris? BMJ Case Rep. 2016;2016:bcr2016214957. 10.1136/bcr-2016-214957 PMC4885247 27151055

[19] Ruiz-Medrano J, Ávalos-Franco N, Gutierrez-Bonet R, Cifuentes-Canorea P, Gegundez-Fernandez JA, Diaz-Valle D (2017). Expulsive total iridodialysis through microincision phacoemulsification wound. J Fr Ophtalmol.

